# Mechanisms of Cognitive Impairment in Cerebral Small Vessel Disease: Multimodal MRI Results from the St George's Cognition and Neuroimaging in Stroke (SCANS) Study

**DOI:** 10.1371/journal.pone.0061014

**Published:** 2013-04-22

**Authors:** Andrew J. Lawrence, Bhavini Patel, Robin G. Morris, Andrew D. MacKinnon, Philip M. Rich, Thomas R. Barrick, Hugh S. Markus

**Affiliations:** 1 Stroke and Dementia Research Centre, St George's University of London, London, United Kingdom; 2 Department of Psychology, Institute of Psychiatry, London, United Kingdom; 3 Department of Neuroradiology, Atkinson Morley Regional Neuroscience Centre, St George's Healthcare NHS Trust, London, United Kingdom; University of Cambridge, United Kingdom

## Abstract

Cerebral small vessel disease (SVD) is a common cause of vascular cognitive impairment. A number of disease features can be assessed on MRI including lacunar infarcts, T2 lesion volume, brain atrophy, and cerebral microbleeds. In addition, diffusion tensor imaging (DTI) is sensitive to disruption of white matter ultrastructure, and recently it has been suggested that additional information on the pattern of damage may be obtained from axial diffusivity, a proposed marker of axonal damage, and radial diffusivity, an indicator of demyelination. We determined the contribution of these whole brain MRI markers to cognitive impairment in SVD. Consecutive patients with lacunar stroke and confluent leukoaraiosis were recruited into the ongoing SCANS study of cognitive impairment in SVD (n = 115), and underwent neuropsychological assessment and multimodal MRI. SVD subjects displayed poor performance on tests of executive function and processing speed. In the SVD group brain volume was lower, white matter hyperintensity volume higher and all diffusion characteristics differed significantly from control subjects (n = 50). On multi-predictor analysis independent predictors of executive function in SVD were lacunar infarct count and diffusivity of normal appearing white matter on DTI. Independent predictors of processing speed were lacunar infarct count and brain atrophy. Radial diffusivity was a stronger DTI predictor than axial diffusivity, suggesting ischaemic demyelination, seen neuropathologically in SVD, may be an important predictor of cognitive impairment in SVD. Our study provides information on the mechanism of cognitive impairment in SVD.

## Introduction

Cerebral small vessel disease (SVD) is the most common pathology underlying vascular dementia, and is a major cause of lesser degrees of vascular cognitive impairment (VCI) [1]. Radiological correlates are lacunar infarcts, with or without more diffuse areas of white matter hyperintensities (WMH), also referred to as leukoaraiosis. Other features are brain atrophy and cerebral microbleeds. Cognitive impairment in SVD is characterised by prominent impairment of executive function and processing speed, with relative preservation of episodic memory [1], [2]. Despite its importance, there are few specific treatments for cognitive impairment in SVD. The development of evidence based treatment approaches depends upon better understanding of the mechanisms of cognitive decline.

A number of potential mechanisms have been suggested, and their role investigated using magnetic resonance imaging (MRI). A popular hypothesis is that white matter damage causes white matter tract disruption and disconnection of cortical-subcortical and cortical-cortical connections underlying complex networks associated with cognitive control mechanisms and efficient information processing [3]. A number of pathologies seen in SVD could potentially cause such disruption including discrete lacunar infarcts, more diffuse regions of leukoaraiosis, and cerebral microbleeds (CMB).

MRI can be used to investigate the role of these different pathologies in causing cognitive impairment. In patients with SVD correlations between T2 lesion volume and cognition are weak [4], [5]. This may reflect the fact that high signal on T2-weighted images represents increased water content and may not differentiate between areas of mildly and severely damaged tissue [6]. Diffusion tensor imaging (DTI) is more sensitive to white matter ultrastructural damage and DTI parameters have been shown to correlate more strongly with cognition than T2 lesion volume [7], [8], [9]. Not only can average diffusivity be measured as a marker of white matter ultrastructure, but the anisotropy of diffusion gives information on its directionality, and thus integrity of white matter structures. It has been recently suggested that additional information obtained from the diffusion tensor can give further insight in the nature of white matter damage. Axial and Radial components of the tensor have been proposed as markers with specificity to the type of neuronal damage [10]. Axial diffusivity (AD) represents diffusivity in the principal diffusion direction (i.e. in the gross orientation of white matter structure) and is a proposed marker of axonal damage, while radial diffusivity (RD), which is the average of diffusivities perpendicular to the principal direction of the tensor (and hence the gross orientation of white matter structure), is proposed to give information on the degree of demyelination. Such information may be relevant in SVD in which both ischaemic demyelination and axonal loss are seen pathologically [1].

Another feature associated with cognitive impairment in SVD is brain atrophy [11], [12]. This could occur due to SVD pathology itself, or grey matter atrophy could occur secondary to white matter tract disruption producing denervation of cortical structures. Most studies in SVD have looked at global brain atrophy rather than differentiating grey and white matter volumes to assess whether changes in one particular brain compartment drive this association. CMB, small deposits of blood product detectable using blood sensitive MRI sequences such has T2*-weighted gradient echo, are also commonly observed in SVD [13] and have been linked to cognitive impairment [14].

Studies conducted to date have frequently looked at one MRI measure in isolation, and most have been in relatively small patient numbers. To investigate the role of these different pathologies, we applied multimodal MRI to a well phenotyped group of patients with SVD and correlated whole brain MRI measures with cognition. Whole brain measures were chosen as they are widely used [15], and technically suited to a role as a clinical marker of SVD where brain changes are anatomically diffuse and variable. We constructed multi-variable regression models to determine the strongest predictors of cognitive function. In addition, we extended upon previous investigations by assessing axial and radial diffusivity.

## Methods

### Subjects

#### (a)SVD patients

Consecutive patients with SVD were recruited to the St George's Cognition and Neuroimaging in Stroke (SCANS) study from stroke services in three hospitals covering a geographically contiguous area of South London (St George's Hospital, King's College Hospital and St Thomas's Hospital). SVD was defined as a clinical lacunar stroke syndrome [16] with an anatomically appropriate lacunar infarct on MRI, as well as confluent leukoaraiosis (Fazekas grade 2 or more) on MRI [17]. All patients were fluent in English to allow neuropsychological testing. Exclusion criteria were: any cause of stroke other than SVD including extra or intracranial arterial vessel stenosis >50%; any cardioembolic source; cortical infarcts; subcortical infarcts >1.5 cm in diameter as these (striatocapsular type infarcts) are often due to embolism; other major central neurological system disorders; major psychiatric disorders (except depression); any cause for white matter disease other than SVD. Individuals with contraindications to MRI including claustrophobia were excluded.

The study was granted ethical approved by Wandsworth REC. 180 patients were screened of whom 137 volunteered to participate and gave written informed consent. 121 of the 137 SVD patients completed the protocol. Of non-completers, 6 withdrew due to the length of the neuropsychology examination, 2 could not complete MRI, 6 became unwell between consenting and testing, and 2 were found to meet exclusion criteria after consent, 1 due to narcolepsy and 1 due to schizophrenia.

All cognitive testing and MRI was performed at least 3 months post-stroke to minimise acute effects of stroke on cognition.

#### (b) Controls

For comparison with the SVD MRI data, a stroke-free control group was used. These were recruited as part of the St George's Neuropsychology and Imaging in Elderly (GENIE) study [18]. Briefly, individuals aged 50–90 at study onset were recruited by random sampling of family doctor lists; all individuals in the UK are registered with a family doctor. Screening ensured subjects were without central nervous system disease including stroke and transient ischaemic attack (TIA). As GENIE is a population study investigating age related change in cognition and MRI, no screening was conducted based on MRI data. MRI scans performed at the 2 year follow-up point were used as these were obtained using the same MRI scanner and sequences used for the SVD group. At this time point MRI data was available for 57 subjects.

### Clinical assessments

All SVD patients were examined by a neurologist. Cerebrovascular risk factors recorded including hypertension, diabetes mellitus, hypercholesterolaemia, body mass index and smoking history as well as current medications. Hypertension was defined as either systolic BP of >140 mmHg, diastolic BP >90 mmHg or on treatment. Hypercholesterolaemia was defined as a random total cholesterol of >5.2 mmol/L or on treatment. Smoking was divided into current, ex-smoker and never. Diabetes mellitus was defined as being on drug or insulin treatment. Disability was assessed using the modified Rankin scale [19].

### MRI Acquisition

Images were acquired using a 1.5-T General Electric Signa HDxt MRI system (General Electric, Milwaukee, WI, USA) with a maximum gradient amplitude of 33 mTm^−1^ and a proprietary head coil. Sequences were acquired across the whole brain and total imaging time was approximately 45 minutes. All subjects were placed in the head coil in a neutral position with an alignment marker at the nasal bridge to standardise head position. Minimal head movement was ensured during the scan by use of foam pads and a Velcro strap.

### Conventional Imaging

1. Axial Fluid Attenuated Inversion Recovery (FLAIR) sequence-TR/TE/TI = 9000/130/2200 ms, field of view (FOV) = 240×240 mm^2^, matrix = 256×192, 28 contiguous slices of 5 mm thickness.
2. Coronal spoiled gradient recalled echo T1-weighted (SPGR) sequence-TR/TE = 11.5/5 ms, FOV = 240×240 mm^2^, matrix = 256×192, Flip Angle = 18°, 176 contiguous slices of 1.1 mm thickness.
3. Gradient Recalled Echo (GRE) T2*-weighted sequence-TR/TE = 300/30 ms, FOV = 240×240 mm^2^, matrix = 256×192, 28 axial slices of 5 mm thickness with no slice gap. Imaging sequences were identical in cases and controls but T2-GRE sequences were not obtained for control subjects.

### Diffusion Tensor Imaging

Axial single shot spin echo planar images (EPI; TE = 93.4 ms, TR = 15600 ms) were acquired to give isotropic voxels and whole brain coverage (2.5 mm^3^; FOV = 240×240 mm^2^, acquisition matrix = 96×96). Following four acquisitions without diffusion weighting (b = 0 s mm^−2^), diffusion sensitised images were acquired with gradients applied (b = 1000 s mm^−2^) in 25 non-collinear directions. This was repeated to acquire a further four b = 0 s mm^−2^ images and the negative of the 25 directions. Images were realigned to remove eddy current distortions using the FSL Linear Image Registration Tool (FLIRT, FMRI Software Library, FSL version 4.1; FMRIB Analysis Group, Oxford, UK, http://www.fmrib.ox.ac.uk/fsl [20]). The geometric average of the positive and negative acquisitions was taken to eliminate gradient cross-terms [21]. The eight b = 0 s mm^−2^ images were coregistered and averaged to give a T2-weighted EPI image (henceforth termed the b0).

### MRI Data analysis

MRI scans were analysed blinded to subject identity.

#### WMH Lesions

White matter hyperintense regions of FLAIR images were delineated by a single rater, using the semi-automated DISPUNC program [22] (David Plummer, University College, London, UK). Within each slice, for each lesion, the user identifies a voxel near the edge of the lesion and an intensity-gradient-based algorithm uses this starting point to automatically delineate the contour of lesion extent. Lesions that were ≥2 mm in diameter were delineated. Whole brain lesion maps were generated and lesion load was then calculated as the percentage of non-normalised brain volume. Brain volume computation techniques are presented below.

#### Lacunar infarcts

A single consultant neuroradiologist evaluated the T1-weighted and FLAIR images for lacunar infarcts, defined as a CSF filled cavity within the white matter or subcortical regions, between 3–15 mm in diameter.

#### Cerebral Microbleeds

Microbleeds were identified on GRE as well-defined focal areas of low signal <10 mm in diameter. Symmetrical areas of basal ganglia calcification, flow voids from blood vessels and low signal due to adjacent bone were discounted. The Brain Observer Microbleed Rating Scale (BOMBS) was used to describe the location of microbleeds [23]. Only microbleeds meeting the “certain” criteria were analysed for this study.

#### Normalised Brain Volume

Normalised Brain Volume (NBV) is an estimate of brain size with respect to skull size and thus a measure of brain atrophy in cross-sectional data [24]. T1-weighted structural images were segmented into tissue probability maps for Grey Matter (GM), White Matter (WM) and Cerebro-Spinal Fluid (CSF) components using the segment function of the Statistical Parametric Mapping (SPM) package (version 8; Wellcome Department of Cognitive Neurology, London; http://www.fil.ion.ucl.ac.uk/spm). GM and WM tissue maps were of the whole brain, with the GM map including both cortical and subcortical tissue. NBV and non-normalised brain volume were then calculated using the automated SIENAX program (FSL version 4.1) [24]. To minimise tissue misclassification due to the presence of lesions, WMH lesion maps were used to correct GM and WM volumes. The correction was carried out by calculating the volume of tissue classified as GM within the WMH region. This figure was then subtracted from the GM volume total and added to the WM volume total. This ensured that hypointense T1-weighted signal in the WM attributable to leukoaraiosis was not misclassified as GM for volume analysis. All tissue segmentations were visually inspected to ensure consistent segmentation results.

#### Diffusion Tensor Histogram Analysis

DTIs were calculated at each voxel from diffusion-weighted images using the log-linear fitting method of Basser et al [25] and Fractional Anisotropy (FA), Mean Diffusivity (MD), AD and RD maps were computed.

To identify normal appearing white matter (NAWM) tissue in DTI space, multimodal coregistration was performed using FLIRT. T1-weighted structural images and T2-weighted FLAIR images were registered to the b0 image using 12 parameter affine registration with a normalised mutual information cost function. These transformations were applied to the tissue probability maps and WMH lesion masks. NAWM in the DTIs was classified as non-lesion voxels where the probability of WM was greater than GM or CSF.

Histograms were calculated for FA, MD, AD and RD maps for NAWM voxels. Each histogram had 100 bins: FA bin width: 0.01, range: 0 to 1. MD, AD and RD bin widths: 4×10^−5^ mm^2^ s^−1^, range: 0 to 4×10^−3^ mm^2^s^−1^. Histogram frequencies were normalised by the total NAWM to correct for individual differences in NAWM volume.

Distributions of FA and diffusivity measures in NAWM are non-Gaussian. For this reason analysis is usually conducted on measures derived from the histogram, therefore histogram measures of Normalised Peak Height (NPH), and Peak Value (or peak location) are preferable to means and standard deviations [8]. Peak values are where DTI measures exhibit maximum frequency. NPH is the proportion of observations at the peak value. We also calculated measures of centrality (mean, median), variability (standard deviation, interquartile range) and distribution shape (skew, kurtosis).

#### Missing MRI Data

Complete MRI scanning data was unavailable in 5 SVD subjects: In four cases excessive geometrical EPI warping was observed in diffusion-weighted images causing co-registration errors. One SVD subject was unable to complete MRI. Complete scanning data was not available for 5 control cases. Of these, two had FLAIR scans which were technically inadequate for WMH lesion volume measurement (excessive movement; acquisition error), one was unable to complete MRI, and two exhibited excessive EPI warping. Consequently complete MRI data were acquired for 115 SVD patients and 50 controls.

### Cognitive Function Assessment

In the SVD group cognitive assessment was performed by a neuropsychologist using a battery of widely-used tasks chosen to characterise the cognitive impairment seen in SVD. Assessment was within 2 weeks of MRI, taking approximately 2.5 hours. Index measures summarising performance across related tasks were produced as detailed in Table S1 in File S1. In addition a Global Cognition index was produced which summarised performance on all the tasks. Premorbid IQ was estimated using the re-standardised National Adult Reading Test (NART; 2^nd^ Edition) [26].

#### Cognitive Index Construction

To construct each index, key measures for each task were transformed into z-scores through psychometric standardisation using the best available age-scaled normative data. An exception was the version of the modified Wisconsin Card Sort Test (mWCST), selected for brevity and sensitivity, for which a published study was used to select normative data with comparable age and gender distribution [27]. Task z-scores were averaged to create the cognitive index scores, obtaining reliable indices (see Results section)

#### Missing Cognitive Data

For missing data where the subject was unable to complete the task, the minimum scaled score attainable for that task was substituted (this was 0 or 1; corresponding to z-scores of −3.33 and −3). If data were missing for other reasons, the cognitive index scores were calculated from the remaining tasks in the index to reduce the cumulative impact of sporadic data loss on sample size. Such missing data comprised 1.2%.

### Statistical Analysis

Data analysis was conducted using R [28]. MRI data were compared between SVD and control groups using t-tests. Repeated measures ANOVA was conducted treating the 6 cognitive indices as a factor to confirm that performance was significantly different across cognitive domains. Custom linear contrasts assessed the a-priori hypothesis that executive function and processing speed would be the most impaired [2], [29], and would therefore show deviation scores significantly different from each of the other 4 indices. One-sample t-tests tested the null-hypothesis that each cognitive index was equal to zero–i.e. that the average performance in the group was equal to the expected performance of age-matched controls.

Within the SVD group, we examined associations between MRI parameters and the key cognitive measures that showed profile based impairment, namely Executive Function and Processing Speed (see Results section). Multiple regression models were used, adjusting for confounder variables: age, gender & premorbid IQ (NART). First, models were constructed for each MRI variable controlling for confounders: “single predictor” models. Second, models including the confounders and all MRI variables were constructed to assess the relative importance of correlated predictors, these are termed “multi-predictor” models. For all models standardised regression coefficients were used to assess relative importance of predictors. Regression results were visually screened for outliers, checked for variance inflation of regression coefficients due to multicollinearity and the quality of overall fit tested with F-tests. Measures of multicollinearity are high when correlated predictors derived from directly related measures are included together in multi-variable models [30]. This is the case for (a) FA, MD, AD and RD histograms, (b) normalised GM, WM and total brain volumes, (c) CMB count and CMB presence variables. To avoid multicollinearity of predictors in the regression model, for each of the three sets above, only the measure with the strongest univariate association was included in the multi-predictor analysis (see Results).

## Results

Final subject numbers for analysis were 115 (SVD) and 50 (Controls). A summary of demographic data for patients and controls is displayed in Table 1.

**Table 1:** Demographics of SVD and control groups.

	SVD Patients (N = 121)	Controls (N = 57)	Group Test Result
Age (years)	70.01 (9.75)	70.36 (9.18)	p<0.82
Gender (% male)	78 (64.5)	35 (62.4)	p<0.25
Hypertension (%)	112 (92.6)	28 (49.1)	p<0.0001
Systolic BP (mmHg)	146.8 (21.47)	138.48 (18.04)	p<0.013
Diastolic BP (mmHg)	80.95 (10.77)	79.27 (12.33)	p<0.36
Cholesterol (mmol)	4.333 (0.899)	5.67 (1.13)	p<0.0001
Diabetes Mellitus (%)	24 (19.8)	0 (0)	p<0.0001
Smoker (current or ex)	55 (45.5)	32 (56.1)	p<0.185
BMI (kg m^−2^)	27.05 (4.88)	25.19 (3.86)	p<0.016

*BP–Blood Pressure; DM–Diabetes Mellitus; BMI–Body Mass Index. All values are Mean (SD) or proportion (%) as specified. Independent sample t-test or Chi-squared analysis used as appropriate.*

### MRI

#### (a) Conventional MRI parameters

Comparisons of MRI parameters between SVD cases and controls are shown in Table 2. NBV was lower in SVD compared to controls (p<0.0001), and this difference appeared to be driven by a reduction in grey matter volumes, which were also significantly lower in the SVD group. WMH lesion load was higher in SVD compared to controls (3.19 v 0.84%, p<0.0001) as was the number of Lacunar infarcts (p<0.0001).

**Table 2:** MRI results in SVD cases and controls.

MRI Measure	SVD	Controls	p value
NBV (whole brain, ml)	1295.1 (91.1)	1337.3 (87.3)	p<0.005
Grey matter normalised vol. (ml)	727.9 (75.4)	792.3 (62.2)	p<0.0001
White matter normalised vol. (ml)	567.1 (71.9)	544.9 (52.0)	p<0.043
WMH lesion vol. (ml)	31.87 (26.97)	8.7 (11.73)	p<0.0001
WMH lesion load (% brain)	3.19 (2.64)	0.84 (1.12)	p<0.0001
Lacune Count	4.26 (5.48)	0.65 (1.51)	p<0.0001
Lacune Count Quartile Descriptives	0,1,2,5,27	0,0,0,1,10	–
CMB Count	4.73 (16.64)	–	–
CMB Count Quartile Descriptives	0,0,0,2,144	–	–

*Values: Mean (Standard Deviation). Quartile descriptives: Minimum, lower quartile, median, upper quartile, maximum; NBV–Normalised Brain Volume, WMH–White Matter Hyperintensity.CMB–Cerebral Microbleed.*

Distributions of lacunar infarct count and CMB count were skewed (Table 2, quartile descriptives), particularly for microbleeds, with most subjects (60%) having no detectable microbleeds. Accordingly these variables were log10 transformed to reduce skew, and for microbleeds the presence of microbleeds was used as a grouping variable to compare those with microbleeds to those without in addition to using the log10 number of microbleeds as a continuous predictor.

#### (b) DTI

**Figure 1:**
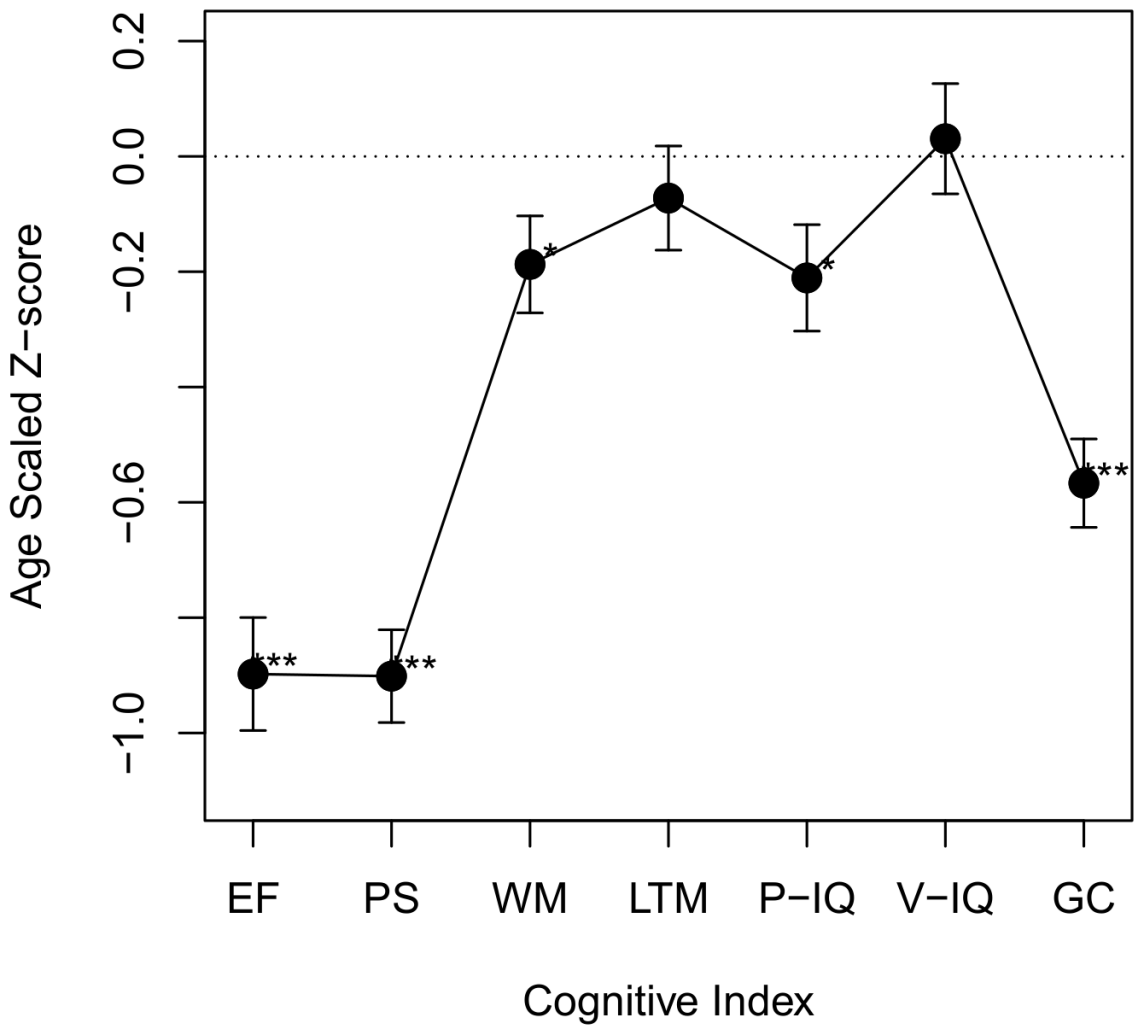
DTI histograms of Normal Appearing WM in SVD and control groups. Normalised frequency histograms of diffusion tensor imaging data are presented for SVD (solid lines) and control (dashed lines) groups. FA–Fractional Anisotropy; MD–Mean Diffusivity (×10^−3^ mm^2^s^−1^); AD–Axial Diffusivity (×10^−3^ mm^2^s^−1^); Radial Diffusivity (×10^−3^ mm^2^s^−1^). SVD–Small Vessel Disease Group. CON–Control Group.

Histograms of FA, MD, AD and RD values in NAWM are displayed in Figure 1. Histogram analysis revealed significant differences between SVD and control groups (Table 3).

**Table 3:** DTI Histogram Group Results.

	SVD (n = 115)	Controls (n = 50)	p-value
Fractional Anisotropy			
Mean	**0.301 (0.0256)**	**0.339 (0.0171)**	**<0.0001**
Std Deviation	0.151 (0.00767)	0.152 (0.00645)	0.3
Skew	**0.693 (0.133)**	**0.545 (0.117)**	**<0.0001**
Kurtosis	**0.421 (0.313)**	**0.172 (0.243)**	**<0.0001**
Peak Location	**0.262 (0.0429)**	**0.311 (0.0294)**	**<0.0001**
Peak Height	0.0272 (0.00213)	0.0269 (0.00196)	0.4
Median	**0.285 (0.0281)**	**0.325 (0.0192)**	**<0.0001**
IQR	0.203 (0.0137)	0.205 (0.0129)	0.4
Mean Diffusivity (×10^−3^ mm^2^s^−1^)			
Mean	**0.870 (0.0442)**	**0.816 (0.0373)**	**<0.0001**
Std Deviation	**0.294 (0.0482)**	**0.250 (0.0366)**	**<0.0001**
Skew	**3.40 (0.593)**	**3.71 (0.779)**	**<0.005**
Kurtosis	**16.5 (5.78)**	**20.7 (7.51)**	**<0.0001**
Peak Location	**0.772 (0.0301)**	**0.750 (0.0319)**	**<0.0001**
Peak Height	**0.140 (0.0212)**	**0.162 (0.0264)**	**<0.0001**
Median	**0.801 (0.0318)**	**0.767 (0.0304)**	**<0.0001**
IQR	**0.176 (0.0353)**	**0.146 (0.0266)**	**<0.0001**
Axial Diffusivity (×10^−3^ mm^2^s^−1^)			
Mean	**1.14 (0.0366)**	**1.12 (0.0366)**	**<0.0001**
Std Deviation	**0.339 (0.0384)**	**0.309 (0.0289)**	**<0.0001**
Skew	**2.43 (0.329)**	**2.31 (0.422)**	**0.044**
Kurtosis	9.26 (2.37)	9.16 (2.69)	0.8
Peak Location	1.02 (0.0324)	1.01 (0.0348)	0.1
Peak Height	0.0827 (0.00619)	0.0844 (0.00637)	0.1
Median	**1.07 (0.0302)**	**1.06 (0.0325)**	**<0.004**
IQR	**0.287 (0.0251)**	**0.275 (0.0230)**	**<0.004**
Radial Diffusivity (×10^−3^ mm^2^s^−1^)			
Mean	**0.732 (0.0506)**	**0.666 (0.0391)**	**<0.0001**
Std Deviation	**0.305 (0.0493)**	**0.259 (0.0371)**	**<0.0001**
Skew	**3.06 (0.529)**	**3.31 (0.703)**	**0.013**
Kurtosis	**14.5 (5.10)**	**18.2 (6.83)**	**<0.0002**
Peak Location	**0.644 (0.0341)**	**0.610 (0.0331)**	**<0.0001**
Peak Height	**0.114 (0.0135)**	**0.127 (0.0147)**	**<0.0001**
Median	**0.670 (0.0371)**	**0.624 (0.0321)**	**<0.0001**
IQR	**0.209 (0.0335)**	**0.180 (0.0242)**	**<0.0001**

Std. Deviation–Standard Deviation; Peak Location–mode of the histogram; Peak Height–normalised frequency of the mode. Test results in bold are significant at p<0.05 level (uncorrected).

1. FA was lower in SVD cases compared with controls. The shape of the distribution differed with increased skew and kurtosis in SVD. Variability of FA values about the mean was comparable: Standard Deviation (SD), peak height and inter-quartile range (IQR) did not differ between groups.
2. MD was higher in SVD cases compared with controls. MD values were also more variable in SVD cases (as shown by SD, IQR, peak height). In contrast to FA histograms, MD histograms were less skewed and less leptokurtic in the SVD group.
3. Differences between SVD cases and controls were greater for RD measures than for AD measures. The pattern of change seen for RD was similar to that for MD.
4. DTI measures were highly correlated, particularly MD which is arithmetically derivable from the AD and RD measures. Correlation coefficients for median diffusion tensor parameters in NAWM are displayed in Table S2 in File S1.

### Cognitive Results

#### Cognitive Index Consistency

Cronbach's Alpha for cognitive indices ranged from 0.69 (Processing Speed) to 0.85 (both Verbal and Performance Intelligence), indicating good internal consistency (see Table S3 in File S1). The Global Cognition index had an alpha of 0.91 indicating very high consistency of general performance across the task battery.

#### Cognitive Profile

**Figure 2:**
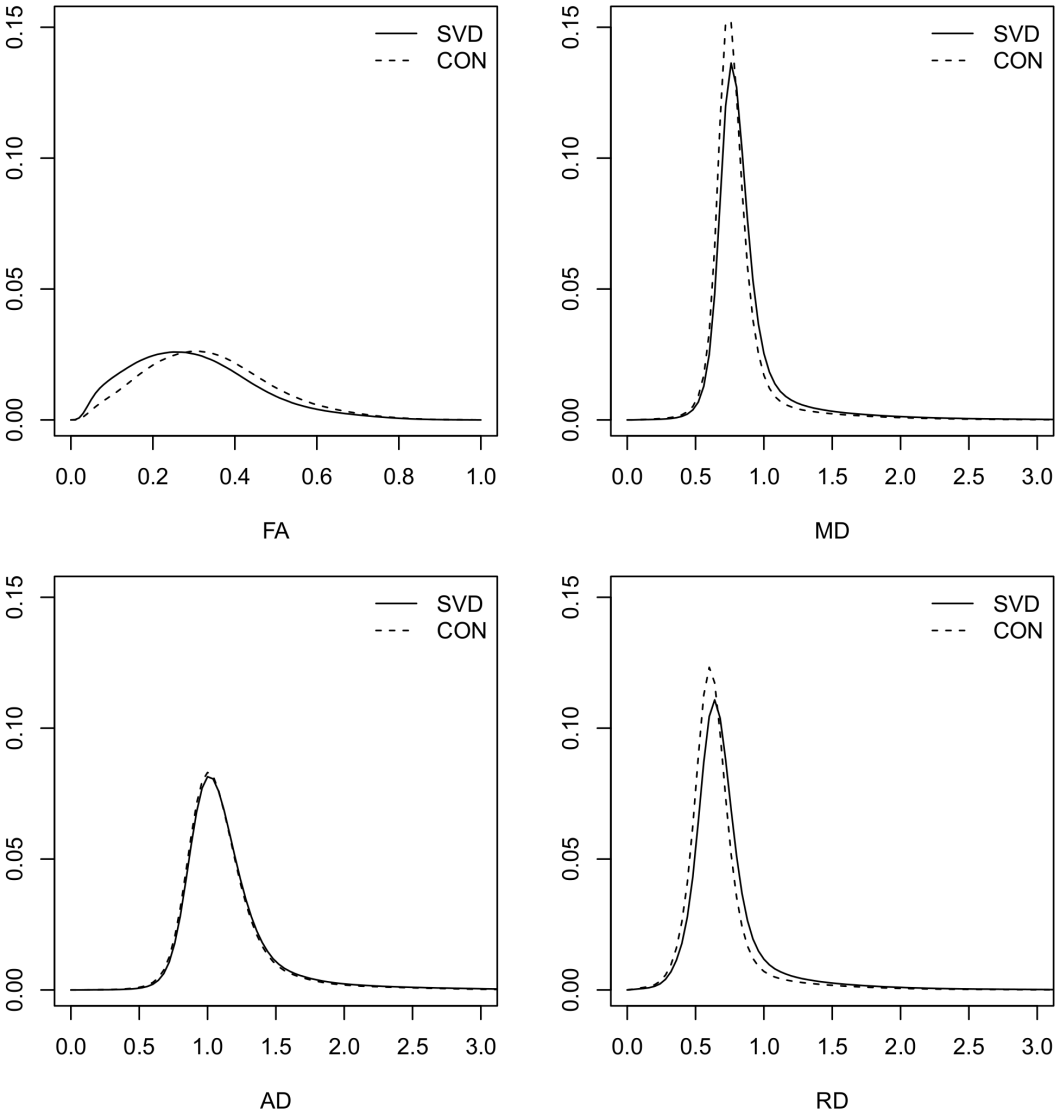
Cognitive Profile in the SVD Group. Average scores for cognitive indices are presented for the SVD group. Error Bars represent +/−1 standard error of the mean. Solid line at zero represents the expected performance for a group of average ability. EF–Executive Function; PS–Processing Speed; WM–Working Memory; LTM–Long-term “episodic” memory; PIQ–Performance IQ; VIQ–Verbal IQ; GC–Global Cognition measure. ***index score significantly different from zero, p<0.001; *index score significantly different from zero, p<0.05.

The profile of cognitive performance in the SVD subjects is shown in Figure 2. There was a significant within-subject effect of cognitive domain (p<0.001). Performance on Executive Function and Processing Speed was poorer than performance on Working Memory (p<0.001), Long-Term Memory (p<0.001), Performance Intelligence (p<0.001) and Verbal Intelligence (p<0.001). No such difference was detectable between Executive Function and Processing Speed (p<0.99). Executive Function and Processing Speed are the cognitive indices primarily impaired in SVD (Pantoni 2010, Zhou & Jia 2009). In our SVD group Working Memory and Performance IQ also significantly differed from expected performance, however, the magnitude of this difference was significantly lower than for Executive Function and Processing Speed. To focus our analysis on the significant determinants of cognitive impairment in SVD we restrict further analysis to the Executive Function and Processing Speed indices.

### Predictors of Executive Function & Processing Speed

#### (a) Single Predictor Models

Regression coefficients between MRI variables and the Executive Function and Processing Speed cognitive measures are shown in Table 4. The pattern of associations with MRI variables was similar for the two cognitive measures. Both NBV and lacunar infarct count were significantly related, along with the volume of grey matter, but not white matter. Lower executive function and processing speed scores were associated with reduced normalised volume (i.e. greater atrophy) and greater numbers of lacunes. Relationships between DTI histogram measures and cognition are shown for FA, MD, AD and RD; all exhibited significant associations. Associations were stronger and more consistent for MD and RD measures, and weaker and less consistent for AD, which did not survive multiple comparisons correction. Overall the strongest association for a diffusion measure was found for the normalised peak height of the RD histogram for Executive Function, and the equivalent MD measure for Processing Speed. In contrast WMH lesion load showed a weak association with Processing Speed which did not survive multiple comparisons correction, and no significant association with Executive Function. Furthermore, neither the number of, nor presence of, microbleeds showed a significant association with the key cognitive measures after correction for multiple comparisons.

**Table 4:** Predictors of key cognitive domains in SVD.

	Single-predictor Models^1^		Multi-predictor Models^2^	
Predictors	EF	PS	EF	PS
NBV (Whole brain)	**.281 (.0001)**	**.387 (<.0001)**	.119 (.25)	**.270 (.0026)**
Normalised GM vol.	.183 (.011)	**.313 (.0001)**	–	–
Normalised WM vol.	.152 (.046)	.140 (.11)	–	–
WMH lesion load (log, %)	−.080 (.25)	−.200 (.013)	.143 (.21)	−.058 (.54)
Lacunar Infarct Count (log)	−**.286 (<.0001)**	−**.355 (<.0001)**	−**.227 (.019)**	−**.244 (.006)**
Cerebral Microbleeds				
Number (log)	−.156 (.026)	−.108 (.21)	−.038 (.6)	.113 (.22)
Presence (Group)	−.042 (.5)	−.059 (.5)	–	–
Fractional Anisotropy				
Histogram Peak Location	**.224 (.002)**	**.268 (.0008)**	–	–
Histogram Peak Height	−.105 (.14)	−.215 (.007)	–	–
Mean Diffusivity (mm^2^s^−1^)				
Histogram Peak Location	−.150 (.035)	−.175 (.031)	–	–
Histogram Peak Height	**.263 (.0002)**	**.281 (.0008)**	**–**	**.085 (0.41)**
Axial Diffusivity (mm^2^s^−1^)				
Histogram Peak Location	−.067 (.4)	−.088 (.29)	–	–
Histogram Peak Height	.184 (.010)	.181 (.030)	–	–
Radial Diffusivity (mm^2^s^−1^)				
Histogram Peak Location	−.175 (.015)	−.204 (.012)	–	–
Histogram Peak Height	**.278 (.0002)**	**.243 (.005)**	**.211 (.046)**	. −

*Values show Standardised regression coefficients: β (p-value) for predictor variables in regression models of: EF-Executive Function, and PS–Processing Speed.*

1 *Single variable models control for effects of age, gender and NART-IQ. Values in Bold remain significant after multiple comparisons correction (Holm-Bonferroni).*

2 *Multiple variable models include all the terms indicated as well as age, gender and NART-IQ. A subset of variables were included in the multi-predictor models, as described in the statistical analysis part of the methods section. Models were highly significant: EF: R^2^ = 0.60; F_(8,105)_ = 19.8, p<0.0001; PS: R^2^ = 0.49; F_(8,105)_ = 12.8, p<0.0001. Significant terms in each model are highlighted in bold.*

#### (b) Multi-predictor Models

Multiple regression was performed to evaluate the relative contribution of the different MRI measures. For highly correlated MRI measures (see statistical methods section), the most significant univariate predictor was included in the multi-predictor model. For Executive Function these were total NBV, CMB count and the normalised peak height of the RD histogram. For Processing Speed, these were total NBV, CMB count and the normalised peak height of the MD histogram. As MD and RD and highly correlated, the above analyses were repeated using MD normalised peak height for Executive Function and RD normalised peak height for Processing Speed. Calculated coefficients were similar (and were identical to 2 decimal places) leading to identical model interpretation.

The regression model (Table 4) provided a highly significant fit for the Executive Function index; the proportion of variance explained was 0.6 (F = 19.8, p<0.00001). The strongest MRI predictor of the Executive Function index was Lacunar infarct count (β = −0.227) followed by the DTI measure: normalised peak height of the white matter radial diffusivity histogram (β = 0.211). The other predictors in the model: NBV, WM lesion load and the presence of microbleeds did not reach significance. Similar results were obtained for the Processing Speed index (R^2^ = 0.5, F = 12.8, p<0.0001), although NBV was now a significant predictor (β = −0.270) and the DTI measure was not (β = 0.085). Post-hoc t-tests based on standardised regression coefficients and pooled variance (analogous to the Hotelling/Williams test for dependent correlation coefficients) were conducted to look for significant differences in the regression coefficients obtained for each predictor in the two cognitive models, none reached significance.

## Discussion

In this well phenotyped cohort of patients with symptomatic small vessel disease and radiological leukoaraiosis, we found a characteristic cognitive profile with impaired performance in executive function and processing speed, relative to working memory and episodic memory. Using a multimodal MRI approach, we investigated the mechanisms underlying this cognitive impairment. On analysis of single predictors, impaired executive function and processing speed were associated with lacunar infarct count, reduced brain volume, and white matter ultrastructural damage determined using DTI. Multi-predictor analysis showed that 60% of variance in executive function and 50% of variance in processing speed was explained by the regression model. For executive function, the strongest predictor was lacunar infarct count followed by the DTI measure. Normalised brain volume and lacunar infarct count were the predictors of processing speed. In these multivariable analyses WMH lesion load, and the presence or number of microbleeds, were not independently associated

Most, but not all, previous studies in sporadic and hereditary (CADASIL) SVD, have found an association between lacunar infarcts and cognition. The independent associations we found with lacunar infarct count may well be mediated by infarcts leading to white matter tract disruption and subsequent cortical-subcortical disconnection. Recent studies using higher magnetic field MRI have shown micro infarcts within the cortex and it is possible that these infarcts, if they associate with total lacunar infarct count, may contribute to this relationship with cognitive impairment [31].

The independent association found for DTI measures supports the role of white matter ultrastructural damage in causing executive dysfunction. Consistent with previous studies [7], [8], including the results of the large multi-centre LADIS study [9], [32], our results show that DTI measures are more sensitive to white matter damage than WMH lesion load. We evaluated a number of markers of the DTI histogram. Associations were generally stronger and more consistent for MD than for FA.

It has been suggested that axial and radial components of the DTI tensor may give further information about the nature of neuronal damage [33] although this has not been previously studied in SVD. AD may represent a marker of axonal damage while RD has been proposed as a marker of demyelination [10]. Both ischaemic demyelination and axonal damage have been reported in neuropathological studies of SVD [34]. Both AD and RD were abnormal in the SVD group relative to controls, but RD was the stronger predictor of executive dysfunction. This may reflect the importance of ischaemic demyelination vs. axonal degeneration in determining the presence of cognitive impairment in SVD, alternatively it may represent relatively greater reliability of the second and third eigenvalues (RD) relative to the first (AD). This analysis has two implications. First, in studies monitoring white matter damage in SVD, RD is a better marker than AD. Second, it provides some support for the role of demyelination in SVD-related cognitive dysfunction.

Previous studies in both sporadic and genetic SVD have reported associations between brain volume and cognition, and in those studies where multimodal MRI has been performed, this association has tended to be independent of the effects of other MRI variables [15], [35]. In this study we found reduced whole brain volume, which was attributable to lower grey matter volume, rather than a reduction in white matter volume. Overall, whole brain volume acted as a better single predictor of cognitive performance than grey matter volume, and in the multi-predictor model a strong independent association was observed between brain volume and processing speed, but this did not reach statistical significance for executive functioning. Although counterintuitive, our finding of WM volume in the high normal range is not necessarily evidence against a key role for demyelination in SVD. While loss of myelin would be expected with volume loss, other white matter pathological processes in SVD including oedema and inflammation, might be associated with volume increase. Of relevance, investigations of WM volume in multiple sclerosis have produced inconsistent findings [36], and this has been explained by the presence of opposing processes (inflammation, gliosis, oedema) acting to increase observed WM volume and counteract the effects of demyelination on WM volume [36], [37].

We found no association between the presence or number of microbleeds and cognitive function. Previous results have produced different conclusions as to the relationship of microbleeds to cognitive dysfunction in SVD [14], [13]. The distribution of microbleeds was skewed with a large number of microbleeds in a number of patients. In a post-hoc analysis [38] we found that a high microbleed count (8 or greater, representing the top 10% of the sample) was related to executive function. However, even the contribution of high levels of microbleeds appears to explain only a small amount of the variation in cognitive function.

### Limitations

The term SVD is used to describe a variety of clinical presentations ranging from asymptomatic white matter hyperintensities in community studies, through to multiple lacunar infarcts and leukoaraiosis in patients with vascular dementia. Furthermore, in patients with symptomatic lacunar stroke due to SVD, it has been suggested there may be more than one underlying pathology. The role of atherosclerosis has been proposed in patients with isolated lacunar infarcts without leukoaraiosis, while a diffuse arteriopathy has been proposed in patients with multiple smaller lacunar infarcts and leukoaraiosis [39], [40]. It is possible the mechanisms causing cognitive impairment in these different situations vary. For this reason we recruited a homogenous group of patients who all presented with symptomatic lacunar infarction and had confluent leukoaraiosis on MRI. This would match with the proposed subtype characterised as secondary to a diffuse arteriopathy.

Axial and radial diffusivity measures, unlike FA and MD, are dependent on eigenvalue sorting and this effect is greater in complex brain tissue such as regions of crossing fibres and pathology [41]. This has implications in interpretation of findings as caution is required in linking AD and RD measures to axonal and demyelinating pathologies in white matter [41]. White matter regions in SVD patients did not show the pattern of changes in AD described as characteristic of axonal damage. Relative to controls, AD was increased, not decreased and this may reflect different disease processes such as tissue oedema, masking the effects of axonal degeneration. Furthermore, MD and RD measures were highly correlated in this sample, and produced interchangeable results in the multivariable models. Although here we address the question of RD and AD as pathological markers separately to the question of the value of conventional MD and FA measures, the extent to which RD and MD provide meaningfully different information about the underlying tissue ultrastructure is unclear. However the results from this baseline analysis suggest they may provide similar information, at least in SVD. This, together with concerns about the validity of the AD–axonal degeneration, and the RD–demyelination link in complex or lesioned WM, suggests further research is required to fully elucidate the relationship between neuropathology and these inter-correlated measurements in SVD.

The whole brain MRI measures presented in this analysis are commonly used [15], and are suited to identifying anatomically diffuse and variable SVD brain changes, however there is mounting evidence that the location of damage in SVD may be important [42], [43], [44], and provide further insight to our understanding of mechanisms in SVD.

Further longitudinal studies correlating MRI changes with cognition are now required. The SCANS dataset described in this paper is the baseline analysis from such a study which is currently performing yearly MRI and cognition over a period of four years.

## Supporting Information

File S1 Supplementary Results Tables. Table S1, Cognitive indices and task measures. Table S2, Inter-individual Median Diffusion Measure Correlations. Table S3, Internal Reliability of Cognitive Indices.
(DOC)
