# Correction: Mechanisms of Cognitive Impairment in Cerebral Small Vessel Disease: Multimodal MRI Results from the St George's Cognition and Neuroimaging in Stroke (SCANS) Study

**DOI:** 10.1371/annotation/bbde462e-c699-4c4d-9b61-050c7e6e5ce3

**Published:** 2013-06-26

**Authors:** Andrew J. Lawrence, Bhavini Patel, Robin G. Morris, Andrew D. MacKinnon, Philip M. Rich, Thomas R. Barrick, Hugh S. Markus

Figures 1 and 2 were incorrectly switched. 

The image appearing as Figure 1 belongs with the label and legend for Figure 2, and the image appearing as Figure 2 belongs with the label and legend for Figure 1. The labels and legends of Figures 1 and 2 are in correct order. 

