# Supplementary material for: Mechanisms of Cognitive Impairment in Cerebral Small Vessel Disease: Multimodal MRI Results from the St George's Cognition and Neuroimaging in Stroke (SCANS) Study
Source: PLoS One. 2013 Apr 22;8(4):e61014. doi: 10.1371/journal.pone.0061014 (PMC3632543; doi:10.1371/journal.pone.0061014)
Supplement: File S1 — Supplementary Results Tables. Table S1, Cognitive indices and task measures. Table S2, Inter-individual Median Diffusion Measure Correlations. Table S3, Internal Reliability of Cognitive Indices. (DOC) [file pone.0061014.s001.doc]

**Supplementary Tables**

***Table S1. Cognitive indices and task measures***

| Cognitive Index | Task & Normative Data reference | Task Measure(s) Used & Additional Details |
| --- | --- | --- |
| Executive Function (EF) | | |
|  |  | [1]Time to complete Part B (number-letter switching) |
|  | Verbal Fluency [2] | Total number of Correct Words generated |
|  | Modified Wisconsin Card Sort Test [3] | Categories Achieved & Perseverative Errors* |
| Processing Speed (PS) | | |
|  | BMIPB Speed of Information Processing [4] | Total correct, adjusted for motor score & errors (%)* |
|  | Digit Symbol Substitution [5] | Total Correct |
|  | Grooved Pegboard Task [6] | Time to complete (average of 2 hands) |
| Working Memory (WM) | | |
|  | Digit Span Task [5] | Total Score |
| Long Term (Episodic) Memory (LTM) | | |
|  | WMS-III Logical Memory [7] | Total Score: Immediate Recall & Delayed Recall* |
|  | WMS-III Visual Reproduction [7] | Total Score: Immediate Recall & Delayed Recall* |
| Performance Intelligence (PIQ) | | |
|  | WASI Block Design [8] | Total Score |
|  | WASI Matrix Reasoning [8] | Total Score |
| Verbal Intelligence (VIQ) | | |
|  | WASI Vocabulary [8] | Total Score |
|  | WASI Similarities [8] | Total Score |

*BMIPB - Birt Memory & Information Processing Battery; WMS-III - Wechsler Memory Scale - Third Edition (UK); WASI - Wechsler Abbreviated Scale of Intelligence. *Composite score used for multiple task measures.*

***Table S2: Inter-individual Median Diffusion Measure Correlations***

*Inter-subject Pearson’s correlations shown for median diffusion tensor measures in normal appearing white matter. All correlations reached high statistical significance, p<0.001. FA – Fractional Anisotropy, AD – Axial diffusivity, RD – Radial Diffusivity, MD – Mean Diffusivity.*

|  | Controls (n=50) | SVD (n=115) | Combined (n=165) |
| --- | --- | --- | --- |
| FA-AD | -0.527 | -0.298 | -0.405 |
| FA-RD | -0.836 | -0.876 | -0.901 |
| FA-MD | -0.746 | -0.755 | -0.804 |
| RD-AD | 0.890 | 0.699 | 0.743 |
| MD-AD | 0.947 | 0.830 | 0.853 |
| MD-RD | 0.987 | 0.975 | 0.981 |

***Table S3***: Internal Reliability of Cognitive Indices

|  | No. Tasks | Valid Cases^a^ | Cronbach's Alpha |
| --- | --- | --- | --- |
| Executive Function (EF) | 3 | 116 | 0.715 |
| Processing Speed (PS) | 3 | 113 | 0.690 |
| Working Memory^b^ (WM) | 1 | - | n/a |
| Long-term Memory (LTM) | 2 | 120 | 0.730 |
| Performance Intelligence (PIQ) | 2 | 121 | 0.848 |
| Verbal Intelligence (VIQ) | 2 | 121 | 0.851 |
| Global Cognition (GC) | 11 | 109 | 0.905 |

*a – The number of valid cases varies with the number of subjects with complete data for* ***all*** *constituent tasks. See missing data paragraph in the methods section.*

*b – Working Memory Index is derived from a single task. Thus reliability can not be computed.*

**Supplementary Citations**

[1] Mitrushina M, Boone KB, Razani J, D’Elia LF (2005) Handbook of Normative Data for Neuropsychological Assessment. USA: Oxford University Press, second edition.

[2] Delis DC, Kaplan E, Kramer JH (2001) Delis-Kaplan Executive Function Scale (D-KEFS). San Antonio, TX: The Psychological Corporation.

[3] Nagahama Y, Okina T, Suzuki N, Matsuzaki S, Yamauchi H, et al. (2003) Factor structure of a modified version of the wisconsin card sorting test: an analysis of executive deficit in Alzheimer’s disease and mild cognitive impairment. Dementia and Geriatric Cognitive Disorders 16: 103–112.

[4] Coughlan AK, Oddy M, Crawford JR (2007) The BIRT Memory and Information Processing Battery (B-MIPB). Wakefield, UK: The Brain Injury Rehabilitation Trust (BIRT).

[5] Wechsler D (1997) Wechsler Adult Intelligence Scale-Third edition (WAIS-III). San Antonio, TX: The Psychological Corporation.

[6] Dawson JD, Uc EY, Anderson SW, Johnson AM, Rizzo M (2010) Neuropsychological predictors of driving errors in older adults. Journal of the American Geriatrics Society 58: 1090–1096.

[7] Wechsler D (1997) Wechsler Memory Scale - Third Edition (WMS-III UK) Administration and Scoring Manual. San Antonio, TX: The Psychological Corporation.

[8] Wechsler D (1999) Wechsler Abbreviated Scale of Intelligence (WASI) Manual. San Antonio, TX: The Psychological Corporation.

Cerebral small vessel disease: from pathogenesis and clinical characteristics to therapeutic
